# Supplementary material for: Systematic review protocol of the effectiveness of HIV prevention interventions for reducing risky sexual behaviour among youth globally
Source: BMJ Open. 2022 May 12;12(5):e056929. doi: 10.1136/bmjopen-2021-056929 (PMC9109094; doi:10.1136/bmjopen-2021-056929)
Supplement: Supplementary data [file bmjopen-2021-056929supp001.pdf]

Supplementary information

Table S1: Search strategy

Theme	Number	Search terms
**participants**	#1	Youth [Text Word] OR Adolescen* [Mesh Term] OR "Young people" [Text Word] OR Teen* [Text Word] OR "Young adults" [Text Word] OR Students [Text Word]
**intervention**	#2	"HIV prevention intervention" [Text Word] OR "HIV prevention strateg*" [Text Word] OR "HIV prevention program*" [Text Word] OR "HIV education prevention program*"[Text Word] OR "Sexual* education program*" [Text Word] OR "Health behav* intervention" [Text Word] OR "Peer* education program*"[Text Word] OR "Peer* HIV prevention intervention" [Text Word] OR "HIV prevention model" [Text Word] OR "HIV/sexually transmitted infection/pregnancy intervention" [Text Word] OR "HIV/AIDS/STI reduction intervention" [Text Word] OR "HIV/STD risk reduction intervention" [Text Word] OR "Behav* intervention" [Text Word] OR "Structural intervention" OR "Behav* intervention" OR "Combin* intervention" OR "Multicomponent intervention" [Text Word] OR "Sexual health intervention" [Text Word] OR "Life skills intervention" [Text Word] OR "HIV/AIDS prevention intervention" [Text Word] OR "HIV/AIDS prevention program*"[Text Word]
**outcomes**	#3	"Risk* sexual behav*" [Text Word] OR "Unsafe sex" [Text Word] OR "High risk* sex" [Text Word] OR "Unhealthy sexual relations" [Text Word] OR "Sexual health behav*" [Text Word] OR "Sexual risk* behav*" [Text Word] OR "HIV/sexually transmitted infection risk behav*" [Text Word] OR "Sexual risk-taking behav*" [Text Word] OR "Risk* behav* practices" [Text Word] OR "Risk* behav*"

		[Text Word] OR "Health risk* behav*" [Text Word] OR "Sexual partners" [Text Word] OR "Sexual behav*" [Text Word] OR "Forced sex" [Text Word] OR "Coerced sex" [Text Word] OR "Early sexual debut" [Text Word] OR "Early marriage" [Text Word] OR "Transactional sex" [Text Word] OR "Intergenerational sex" [Text Word] OR Multiple sexual partners [MeSH term] OR "Sex partners" [Text Word] OR "Multiple sex partners" OR "Concurrent sexual partners" [Text Word] OR "Condomless sex" [Text Word] OR "Unprotected sex" [Text Word] OR "Inconsistent condom use" [Text Word] OR "Pregnancy incidence" [Text Word]
**Search**	#4	1 AND 2 AND 3 = (Youth [Text Word] OR Adolescen* [Mesh Term] OR "Young people" [Text Word] OR Teen* [Text Word] OR "Young adults" [Text Word] OR Students [Text Word]) AND ("HIV prevention intervention" [Text Word] OR "HIV prevention strateg*" [Text Word] OR "HIV prevention program*" [Text Word] OR "HIV education prevention program*"[Text Word] OR "Sexual* education program*" [Text Word] OR "Health behav* intervention" [Text Word] OR "Peer* education program*"[Text Word] OR "Peer* HIV prevention intervention" [Text Word] OR "HIV prevention model" [Text Word] OR "HIV/sexually transmitted infection/pregnancy intervention" [Text Word] OR "HIV/AIDS/STI reduction intervention" [Text Word] OR "HIV/STD risk reduction intervention" [Text Word] OR "Behav* intervention" [Text Word] OR "Structural intervention" OR "Behav* intervention" OR "Combin* intervention" OR "Multicomponent intervention" [Text Word] OR "Sexual health intervention" [Text Word] OR "Life skills intervention" [Text Word] OR "HIV/AIDS

		prevention intervention" [Text Word] OR "HIV/AIDS prevention program*"[Text Word]) AND "Risk* sexual behav*" [Text Word] OR "Unsafe sex" [Text Word] OR "High risk* sex" [Text Word] OR "Unhealthy sexual relations" [Text Word] OR "Sexual health behav*" [Text Word] OR "Sexual risk* behav*" [Text Word] OR "HIV/sexually transmitted infection risk behav*" [Text Word] OR "Sexual risk-taking behav*" [Text Word] OR "Risk* behav* practices" [Text Word] OR "Risk* behav*" [Text Word] OR "Health risk* behav*" [Text Word] OR "Sexual partners" [Text Word] OR "Sexual behav*" [Text Word] OR "Forced sex" [Text Word] OR "Coerced sex" [Text Word] OR "Early sexual debut" [Text Word] OR "Early marriage" [Text Word] OR "Transactional sex" [Text Word] OR "Intergenerational sex" [Text Word] OR Multiple sexual partners [MeSH term] OR "Sex partners" [Text Word] OR "Multiple sex partners" OR "Concurrent sexual partners" [Text Word] OR "Condomless sex" [Text Word] OR "Unprotected sex" [Text Word] OR "Inconsistent condom use" [Text Word] OR "Pregnancy incidence" [Text Word]
**Limits**	#5	Publications from 2011/08/01 to 2021/08/31<br>Age outside 15 to 24 years<br>Non-English publications
