# Supplementary material for: Systematic review protocol of the effectiveness of HIV prevention interventions for reducing risky sexual behaviour among youth globally
Source: BMJ Open. 2022 May 12;12(5):e056929. doi: 10.1136/bmjopen-2021-056929 (PMC9109094; doi:10.1136/bmjopen-2021-056929)
Supplement: Author's manuscript [file bmjopen-2021-056929.draft_revisions.pdf]

BMJ Open

BMJ Open is committed to open peer review. As part of this commitment we make the peer review history of every article we publish publicly available.

When an article is published we post the peer reviewers' comments and the authors' responses online. We also post the versions of the paper that were used during peer review. These are the versions that the peer review comments apply to.

The versions of the paper that follow are the versions that were submitted during the peer review process. They are not the versions of record or the final published versions. They should not be cited or distributed as the published version of this manuscript.

BMJ Open is an open access journal and the full, final, typeset and author-corrected version of record of the manuscript is available on our site with no access controls, subscription charges or pay-per-view fees (http://bmjopen.bmj.com).

If you have any questions on BMJ Open's open peer review process please email info.bmjopen@bmj.com

BMJ Open

A systematic review protocol of the effectiveness of HIV prevention interventions for reducing risky sexual behaviour among youth globally

Journal:	*BMJ Open*
Manuscript ID	bmjopen-2021-056929
Article Type:	Protocol
Date Submitted by the Author:	01-Sep-2021
Complete List of Authors:	Mbengo, Fungai; Edith Cowan University, School of Nursing and Midwifery<br>Zgambo, Maggie; Edith Cowan University, School of Nursing and Midwifery<br>Afrifa-Yamoah, Ebenezer; Edith Cowan University, School of Science<br>Kalembo, Fatch; Curtin University, School of Nursing, Midwifery and Paramedicine<br>Honda, Takanori ; Kyushu University, Department of Epidemiology and Public Health, Graduate School of Medical Sciences<br>Shinpuku, Yoko ; Hiroshima University, Department of Global Health Nursing, Division of Nursing Sciences, Graduate School of Biomedical and Health Sciences<br>Chen, Sanmei; Hiroshima University, Department of Global Health Nursing, Division of Nursing Sciences, Graduate School of Biomedical and Health Sciences
Keywords:	HIV & AIDS < INFECTIOUS DISEASES, Public health < INFECTIOUS DISEASES, Infection control < INFECTIOUS DISEASES

SCHOLARONE™
Manuscripts

A systematic review protocol of the effectiveness of HIV prevention interventions for reducing risky sexual behaviour among youth globally

Fungai Mbengo[1], Maggie Zgambo[1], Ebenezer Afrifa-Yamoah[2], Fatch Welcome Kalembo[3], Takanori Honda[4], Yoko Shimpuku[5], Sanmei Chen[†5]

Affiliations:

[1] School of Nursing and Midwifery, Edith Cowan University, Western Australia, Australia

[2] School of Science, Edith Cowan University, Western Australia, Australia

[3] School of Nursing, Midwifery and Paramedicine, Curtin University, Western Australia, Australia

[4] Department of Epidemiology and Public Health, Graduate School of Medical Sciences, Kyushu University, Fukuoka, Japan

[5] Department of Global Health Nursing, Division of Nursing Sciences, Graduate School of Biomedical and Health Sciences, Hiroshima University, Hiroshima Japan

[†]Corresponding author:

Dr. Sanmei Chen

Department of Global Health Nursing, Division of Nursing Sciences,

Graduate School of Biomedical and Health Sciences, Hiroshima University

734-8553, 1-2-3 Kasumi, Minami Ward, Hiroshima City, Japan

For peer review only

TEL: +8182-257-5377 Email: chens@hiroshima-u.ac.jp

ORCID: 0000-0003-0811-1701

Authors' email address:

Fungai Mbengo: fmbengo@our.ecu.edu.au

Maggie Zgambo: m.zgambo@ecu.edu.au

Ebenezer Afrifa-Yamoah: e.afrifayamoah@ecu.edu.au

Fatch Welcome Kalembo: fatchwelcom.kalembo@curtin.edu.au

Takanori Honda: honda.takanori.597@m.kyushu-u.ac.jp

Yoko Shimpuku: yokoshim@hiroshima-u.ac.jp

Sanmei Chen: chens@hiroshima-u.ac.jp

For peer review only

For peer review only - http://bmjopen.bmj.com/site/about/guidelines.xhtml

ABSTRACT

Objective To synthesise current global evidence on the effectiveness of HIV prevention interventions for reducing risky sexual behaviour among youth in the last decade.

Design Systematic review and meta-analysis

Data sources The following databases will be searched for articles published from August 2011 to August 2021: MEDLINE/PubMed, EMBASE, PsychINFO, ProQuest Central, CINAHL and Web of Science databases.

Eligibility criteria for selecting studies Randomised control trials or quasi-experimental studies that examined the effectiveness of HIV prevention interventions among youth populations (15-24 years) with risky sexual behaviour as a primary or secondary outcome.

Data extraction and synthesis Reviewers will assess eligibility, extract data and appraise articles independently. Covidence online systematic review tool will be used to select articles while Joanna Briggs Institute (JBI) data extraction tool will be used to abstract data. Risk bias and quality of evidence of individual studies will be assessed by the Critical Appraisal Checklist for Randomized Controlled Trials and JBI Critical Appraisal Checklist for Quasi-Experimental Studies. The quality of evidence for each outcome will be assessed by the Grading of Recommendations Assessment, Development, and Evaluation (GRADE) approach. Data analysis will be undertaken using RevMan software version 5.3.3. A random effects meta-analysis will be conducted to report heterogeneous data where statistical pooling is achievable. We will use I^2

For peer review only - http://bmjopen.bmj.com/site/about/guidelines.xhtml

statistics to test for heterogeneity. Where appropriate, a funnel plot will be generated to assess publication bias. Where statistical pooling is unachievable, the findings will be reported in a narrative form, together with tables and figures to assist in data presentation if required. Reporting of this systematic review will follow the Preferred Reporting Items for Systematic Reviews and Meta-Analyses (PRISMA) guidelines.

Conclusion Our systematic review has a potential to advance findings and efforts by researchers in different regions of the world by synthesising recent global evidence on the topic.

Keywords effectiveness; HIV prevention intervention; risky sexual behaviour; systematic review protocol; youth

For peer review only

For peer review only - http://bmjopen.bmj.com/site/about/guidelines.xhtml

Strengths and limitations of this study

- This systematic review protocol reduces the possibility of duplication and follows the Preferred Reporting Items for Systematic Review and Meta-analysis Protocols guidelines with transparency to the methods and processes that will be used.

- This systematic review will address a gap in the current global evidence by comprehensively including research findings worldwide on the effectiveness of HIV prevention interventions for reducing risky sexual behaviour among youth in the last decade.

- This systematic review will be one of the few to focus on not only behavioural-level, but also structural-level and combined interventions for HIV prevention targeting youth.

- This systematic review will not search non-English electronic databases. This limitation will result in language bias.

- There is a potential for a small number of studies that used behavioural-level interventions, which may limit the statistical power in the data synthesis for subgroup analyses.

For peer review only - http://bmjopen.bmj.com/site/about/guidelines.xhtml

INTRODUCTION

According to the Joint United Nations Programme on HIV/AIDS (UNAIDS), 38 million people were living with Human Immunodeficiency Virus (HIV) in 2019 and about 35 million had died since HIV was first recognized globally.[1] Over the last decade, there has been a remarkable reduction in HIV infections, HIV related mortality and morbidity in the general population globally following the introduction of the universal antiretroviral therapy.[2] However, recent reports indicate that HIV remains a leading cause of death among the youth population across sub-Saharan Africa, where 80% of young people living with HIV reside.[1 3] Furthermore, in 2019, one third of the global HIV infections occurred among youth aged between 15 and 24 years and the infection rate in this population is anticipated to increase yearly by 13% leading to an estimation of 3.5 million new infections by 2030.[4 5]

Youth are vulnerable to HIV because of a myriad of contextual factors that influence risky sexual behaviours including low self-esteem, poverty, peer pressure[6], alcohol or drug abuse[7], cultural practices, gender-disparities[8 9], limited HIV-related knowledge and gender-based violence.[10] According to the socio-ecological model, these contextual factors affect youth at individual, interpersonal, community, institutional and structural levels.[11 12] This model has been used to guide the development of HIV prevention interventions aimed at reducing risky sexual behaviours among youth .[12-15]

HIV prevention interventions for reducing risky sexual behaviours can be classified as either structural, behavioural or combined.[13-15] Structural interventions aim to reduce risky sexual behaviour by addressing structural-level factors such as poverty,

For peer review only - http://bmjopen.bmj.com/site/about/guidelines.xhtml

unemployment, limited access to education and social norms.[13-15] The behavioural interventions facilitate risky sexual behaviour reduction by targeting individual-level factors such as knowledge, attitudes, self-esteem and self-efficacy.[13-15] Lastly, the combined interventions reduce risky sexual behaviour by addressing one or more individual-level factors, as well as one or more structural-level factors.[13-15]

In the last decade, researchers have conducted systematic reviews on structural, behavioural or combined HIV prevention interventions targeting youth.[13 16-21] However, these reviews have synthesised research findings from studies that were conducted in a specific region, for instance, Sub-Saharan Africa[13 16 17], developing countries[18 19], middle-income countries[20], or a specific country.[21] One review has particularly focused on the female gender.[13] Most of these reviews have focused on behavioural interventions only.[16 18-20] Synthesising global evidence on the subject has potential to identify more effective interventions for reducing risky sexual behaviours among youth.

Following this background, we propose to undertake a systematic review and meta-analysis to synthesise current global evidence on the effectiveness of HIV prevention interventions for reducing risky sexual behaviours among youth in the last decade. Specifically, this systematic review and meta-analysis seeks to answer the following questions:1) What HIV prevention interventions are effective for reducing risky sexual behaviour among youth? 2) What is the level of effectiveness of interventions designed to reduce risky sexual behaviour among youth? 3) What factors affect the effectiveness of the identified interventions? To prevent duplication of reviews, a

For peer review only - http://bmjopen.bmj.com/site/about/guidelines.xhtml

For peer review only

preliminary search of similar protocols or reviews was conducted in July 2021 in CINAHL, Cochrane Library, MEDLINE, Google Scholar, The International Prospective Register of Systematic Reviews (PROSPERO) and Joanna Briggs Institute (JBI) databases. No review protocol or systematic review on this topic published in the last decade was identified.

METHODS

This will be a systematic review and meta-analysis designed to analyse recent global evidence on the effectiveness of HIV prevention interventions for reducing risky sexual behaviour among youth. This review will commence from August 2021 and is expected to complete in February 2022. This protocol has been developed in accordance with the Preferred Reporting Items for Systematic Review and Meta-analysis Protocols (PRISMA-P) guidelines.[22] Reporting of the synthesised findings will utilise the PRISMA guidelines.[23] This protocol is under review for registration with PROSPERO. Important amendments to this protocol will be published along with the results of the systematic review.

Eligibility criteria

To be eligible for this review, studies will need to report on youth aged from 15 to 24 years and be conducted in either clinical or non-clinical setting. Inclusion of studies with participants aged within and outside the identified age bracket will depend on the mean age that falls within the age range of 15-24 years. For recency, all studies conducted from August 2011 to August 2021 will be considered. The review will include studies

For peer review only

For peer review only - http://bmjopen.bmj.com/site/about/guidelines.xhtml

conducted in any geographical location. This review will include the following 3 types of intervention: 1) behavioural interventions that facilitate reduction of risky sexual behaviour by addressing individual-level factors; 2) structural interventions that facilitate reduction of risky sexual behaviour by targeting structural-level factors; 3) combined interventions that facilitate reduction of risky sexual behaviour by targeting at least one individual-level factor and at least one structural-level factor. Studies that do not measure risky sexual behaviour as a primary or secondary outcome will be excluded. In the source trials, the comparators could be control groups who received no intervention or alternative usual interventions, or waitlist controls. The review will include studies conducted using experimental designs such as quasi-experimental, randomized controlled trials and cluster-randomized trials and non-randomized controlled trials. Only studies conducted in English will be considered.

Information sources

We will electronically search the following databases to retrieve relevant articles: MEDLINE/PubMed, EMBASE, PsychINFO, ProQuest Central, CINAHL and Web of Science databases. We will also search ClinicalTrials.gov and the World Health Organization International Clinical Trials Registry Platform to identify ongoing or unpublished eligible trials. To maximise the search for relevant articles, we will review reference lists of included trials and systematic reviews conducted on effectiveness of HIV prevention interventions for reducing risky sexual behaviour among youth.

For peer review only

For peer review only - http://bmjopen.bmj.com/site/about/guidelines.xhtml

Search strategy

The Population, Intervention, Comparator and Outcome (PICO) Model as recommended by JBI will be used to develop a search grid for this review. In this review, the participants are youth aged from 15 to 24 years of age; interventions are HIV prevention interventions; comparators are control groups, and outcomes are risky sexual behaviours. The search grid with identified PICO concepts has been illustrated in Table 1.

Using the identified PICO concepts, a three-step search strategy will be utilised to identify relevant studies. First, keywords for PICO concepts will be brainstormed by reviewers before undertaking an initial limited search of PICO concepts in the MEDLINE/PubMed database to identify controlled vocabulary Medical Subject Headings (MeSH). A second search of EMBASE, MEDLINE/PubMed, PsychINFO, ProQuest Central, CINAHL and Web of Science databases will be conducted using the identified keywords and MeSH terms. Third, we will use these databases to find other relevant keywords and terms. Boolean operators such as 'OR' and 'AND' will be applied when combining similar search terms and different search terms, respectively. For example, (effectiveness OR effects OR outcomes OR impact OR efficacy) AND (youth OR young people OR teen OR young adults OR adolescents). Preliminary search syntax for MEDLINE/PubMed database are included in Table 2.

For peer review only - http://bmjopen.bmj.com/site/about/guidelines.xhtml

Study Records

Data management and selection process

Following the search, all identified citations will be collated and uploaded into EndNote X9 Reference Management System. Duplicates will be removed before importing the references into the Covidence online systematic review tool[24] as recommended by the Cochrane Collaboration. This tool is designed to assist reviewers to screen abstracts and full texts of identified articles, risk of bias assessment and data extraction.[24] In this review, however, this tool will only be used to select eligible studies. Abstracts of the relevant full texts will be assessed for eligibility by FM, MZ and SC, independently. Full-text articles for the selected titles will be further reviewed independently by these reviewers. Each reviewer will leave an audit trail with reasons for each decision undertaken, which will later be compared across all the reviewers. Any disagreements that arise among the reviewers at each stage of the study selection process will be resolved through discussions to reach a consensus. The results of the search will be illustrated following the PRISMA flow diagram.[23]

Assessment of risk of bias and quality of evidence

Three reviewers, FM, MZ and SC will independently assess the quality of each included study using the JBI Critical Appraisal Checklist for Randomized Controlled Trials and JBI Critical Appraisal Checklist for Quasi-Experimental Studies.[25] Reviewers will discuss to agree on dissonant findings from the quality assessment of articles. Following the critical appraisal, studies that do not meet the 50% quality threshold will be excluded.

For peer review only

We will also examine the quality of evidence for each outcome by using the GRADE approach [26], because the quality of evidence often varies between outcomes.[27] Authors of papers will be contacted to request missing information that is necessary for appraisal.

Data extraction process

Data will be extracted by FM using the standardised JBI data extraction tool.[25] MZ and SC will verify the extracted data. Details extracted will include: 1) characteristics of the study: author, year of publication, the title of the study, study objective, study location, setting, 2) methodological characteristics: study design, research questions and/or hypotheses, study population, sample characteristics, groups and controls, type of intervention, length of intervention, delivery mode, theoretical framework, and length of follow-up, measurements, data analyses; 3) main findings and conclusions. Authors will be contacted to request for full articles if only abstracts are accessible and for information if the main outcome data and methods are missing or unclear.

Outcomes and prioritisation

The primary outcome of this review will be risky sexual behaviour, which will be defined as engaging in one or more of the following sexual behaviours: 1) having first sexual activity at or before the age of 15 (early sexual debut), 2) engaging in sexual activity frequently, 3) engaging in sexual activity without a condom, 4) not using condoms consistently, 5) having multiple and/or concurrent sexual partners, 6) having a sexual partner who is five years and older (intergenerational sex), 7) engaging in sexual activity

For peer review only - http://bmjopen.bmj.com/site/about/guidelines.xhtml

under the influence of drugs or alcohol, 8) engaging in sexual activity in exchange for money or gifts (transactional sex), 9) forced to have sexual activity and 10) having been pregnant or fathered a child. [28 29] The secondary outcome measures will be sexually transmitted diseases (STDs) rates, pregnancy rates, birth rates and changes in mediating factors such as knowledge, attitudes, and beliefs that affect risky sexual behaviours. Factors that affect the effectiveness of the intervention such as study design, sample characteristics, intervention content, duration of intervention, the dosage of intervention, length of follow-up and theoretical framework will also be examined.

Data synthesis

Statistical analyses will be conducted using RevMan software (version 5.3.3; The Cochrane Collaboration). We will perform analyses for all outcomes on an intention to treat basis. Effect sizes expressed as odds ratio (for dichotomous data) and weighted mean differences or standardised mean differences (for continuous data) and their 95% confidence intervals will be calculated for analysis. A *p*-value of less than 0.05 will be considered as statistically significant. Heterogeneity will be assessed statistically using the Chi-squared test and the I-squared index.[30] We will use fixed effects models to pool outcomes if significant heterogeneity is not present (I^2 <50%). We will use random effects models when significant heterogeneity is present (I^2 ≥50%). If sufficient data is available, subgroup analyses will be conducted to explore the heterogeneity between the studies. A funnel plot will be generated to assess publication bias if there are 10 or more studies included in a meta-analysis. Statistical tests for funnel plot asymmetry (Egger test, Begg test, Harbord test) will be performed where appropriate. We will

For peer review only - http://bmjopen.bmj.com/site/about/guidelines.xhtml

analyse the data separately for each of the 3 types of interventions (i.e., structural, behavioural, or combined interventions). Several subgroup analyses will be conducted based on study setting, year of publication, length of follow-up and so on. We will also conduct sensitivity analyses to test the robustness of our findings, such as by excluding quasi-randomised trials, excluding trials with high or unknown risk of bias. Where statistical pooling is unachievable[31], the findings will be reported in a narrative form, together with tables and figures to assist in data presentation if required.

Conclusion

This protocol proposes a systematic review to examine the effectiveness of HIV prevention interventions for reducing risky sexual behaviour among youth (15-24 years) throughout the world. HIV prevention interventions remain an important strategy to reduce HIV incidence among youth as a cure for HIV is yet to be discovered. Identifying an effective intervention approach has the potential to provide significant health gains by reducing HIV incidence. The findings of the study will be of interest to researchers, practitioners, policy makers and other healthcare experts.

For peer review only

For peer review only - http://bmjopen.bmj.com/site/about/guidelines.xhtml

Competing interest

We have no competing interests to declare.

Author contributions

FM, MA, and SC conceptualized and designed the protocol, and drafted the manuscript. EAY, FWK, TH and YS contributed to the plan of search strategy, data extraction, and data analysis. All authors contributed to the manuscript revision.

Study funding

SM was supported by Grants-in-Aid for Early-Career Scientists (JP19K19474) from the Ministry of Education, Culture, Sports, Science and Technology of Japan. The funding source had no role in the study conception, the preparation of the manuscript, or the decision to submit the manuscript for publication.

For peer review only - http://bmjopen.bmj.com/site/about/guidelines.xhtml

References

1. UNAIDS AIDSinfo: people living with HIV receiving ART. https://aidsinfo.unaids.org/ (accessed 6 Jun 2021).
2. Teeraananchai S, Kerr S, Amin J, et al. Life expectancy of HIV-positive peopleafter starting combination antiretroviral therapy: a meta-analysis. *HIV medicine* 2017;18(4):256-66. doi:10.1111/hiv.12421.
3. Kharsany ABM, Karim QA. HIV Infection and AIDS in Sub-Saharan Africa: Current Status, Challenges and Opportunities. *The open AIDS journal* 2016;10:34-48. doi:10.2174/1874613601610010034.
4. United Nations Children's Fund. Children and AIDS. Statistical Update. (2017).
5. UNAIDS. AIDSinfo: Global data on HIV epidemiology and response http://aidsinfo.unaids.org/ (accessed 29 Aug 2021).
6. DiClemente RJ, Crittenden CP, Rose E, et al. Psychosocial predictors of HIV-associated sexual behaviors and the efficacy of prevention interventions in adolescents at-risk for HIV infection: what works and what doesn't work? *Psychosomatic Medicine* 2008;70(5):598-605. doi:10.1097/PSY.0b013e3181775edb.
7. Govender K, Cowden RG, Asante KO, et al. Sexual risk behavior: A multi-system model of risk and protective factors in South African adolescents. *Prevention Science* 2019;20(7):1054-65. doi:10.1007%2Fs11121-019-01015-3.
8. Dancy BL, Jere DL, Kachingwe SI, et al. HIV risk reduction intervention for rural adolescents in Malawi. *Journal of HIV/AIDS & social services* 2014;13(3):271-91. doi:10.1080/15381501.2013.864173.

For peer review only

For peer review only - http://bmjopen.bmj.com/site/about/guidelines.xhtml

9. Van Staden A, Badenhorst G. Reviewing gender and cultural factors associated with HIV/AIDS among university students in the South African context. *Curationis* 2009;*32*(4):19-28.

10. Harper GW, Riplinger AJ, Neubauer LC, et al. Ecological factors influencing HIV sexual risk and resilience among young people in rural Kenya: Implications for prevention. *Health education research* 2014;29(1):131-46. doi: 10.1093/her/cyt081.

11. Khuzwayo N,Taylor M. Exploring the socio-ecological levels for prevention of sexual risk behaviours of the youth in uMgungundlovu District Municipality, KwaZulu-Natal. *African journal of primary health care & family medicine* 2018;10(1):e1-e8. doi:10.4102/phcfm.v10i1.1590.

12. Lee BC, Bendixsen C, Liebman AK, et al. Using the Socio-Ecological Model to Frame Agricultural Safety and Health Interventions. *Journal of Agromedicine* 2017;22(4):298-303. doi:10.1080/1059924X.2017.1356780.

13. Muthoni CN, Kneipp SM, Gichane MW, et al. A systematic review of HIV interventions for young women in sub-Saharan Africa. *AIDS and Behavior* 2020;24:3395-413. doi:10.1007/s10461-020-02914-1.

14. Pettifor A, Bekker LG, Hosek S, et al. Preventing HIV among young people: research priorities for the future. *Journal of acquired immune deficiency syndromes (1999)* 2013;63(0 2):S155. doi:10.1097/QAI.0b013e31829871fb.

15. Hosek S, Pettifor A. HIV Prevention Interventions for Adolescents. *Current HIV/AIDS reports* 2019;16(1):120-8. doi:10.1007/s11904-019-00431-y.

For peer review only - http://bmjopen.bmj.com/site/about/guidelines.xhtml

16. Mwale M, Muula AS. Systematic review: a review of adolescent behavior change interventions [BCI] and their effectiveness in HIV and AIDS prevention in sub-Saharan Africa. *BMC public health* 2017;17(1):1-9. doi:10.1186/s12889-017-4729-2.
17. Sani AS, Abraham C, Denford S, et al. School-based sexual health education interventions to prevent STI/HIV in sub-Saharan Africa: a systematic review and meta-analysis. *BMC public health* 2016;16(1):1-26. doi:10.1186/s12889-016-3715-4.
18. Dei Jnr LA. The efficacy of HIV and sex education interventions among youths in developing countries: A review. *Scientific & Academic Publishing* 2016:6(1): 1-17. doi:10.5923/j.phr.20160601.01.
19. Ibrahim S, Sidani S. Community based HIV prevention intervention in developing countries: a systematic review. *Advances in Nursing* 2014;2014. doi:10.1155/2014/174960.
20. Fonner VA, Armstrong KS, Kennedy CE, et al. School Based Sex Education and HIV Prevention in Low and Middle-Income Countries: A Systematic Review and Meta-Analysis. *PLOS ONE* 2014;9(3):e89692. doi:10.1371/journal.pone.0089692.
21. Scott-Sheldon AJL, Walstrom P, Harrison AC, et al. Sexual risk reduction interventions for HIV prevention among South African Youth: a meta-analytic review. *Current HIV research* 2013;11(7):549-58. doi:10.2174/1570162x12666140129105726.

For peer review only - http://bmjopen.bmj.com/site/about/guidelines.xhtml

22. Shamseer L, Moher D, Clarke M, et al. Preferred reporting items for systematic review and meta-analysis protocols (PRISMA-P) 2015: elaboration and explanation. *BMJ* 2015;349(jan02 1):g7647. doi:10.1136/bmj.g7647.

23. Moher D, Liberati A, Tetzlaff J, et al. Preferred reporting items for systematic reviews and meta-analyses: the PRISMA statement. *PLoS medicine* 2009;6(7):e1000097. doi:10.1371/journal.pmed.1000097.

24. Coevidence. How to conduct a systematic review from beginning to end. https://www.covidence.org/blog/how-to-conduct-a-systematic-review-from beginning-to end/?campaignid=13271466385&adgroupid=123024099579&adid=524233276792&gclid=Cj0KCQjw0KHBhDDARIsAFJ6UGg4C_yy8JcRM0TnV0yUnCjPInKnkngt39SX10gyJ_Xyszbo9SWyfQYaAmTWEALw_wcB (accessed 12 Jul 2021).

25. Aromataris E, Munn Z, eds. Joanna Briggs Institute Reviewer's Manual [Internet]. Adelaide: Joanna Briggs Institute. https://reviewersmanual.joannabriggs.org/ (accessed 26 April 2020).

26. Guyatt GH, Oxman AD, Vist GE, et al. GRADE: an emerging consensus on rating quality of evidence and strength of recommendations. *BMJ* 2008;336:924-6. doi:10.1136/bmj.39489.470347.AD.

27. Balshem H, Helfand M, Schunemann HJ, et al. GRADE guidelines: 3. Rating the quality of evidence. *Journal of clinical epidemiology*. 2011;64(4):401-6. doi:10.1016/j.jclinepi.2010.07.015.

For peer review only - http://bmjopen.bmj.com/site/about/guidelines.xhtml

28. Brawner BM, Alexander KA, Fannin EF, et al. The Role of Sexual Health Professionals in Developing a Shared Concept of Risky Sexual Behavior as it Relates to HIV Transmission. *Public health nursing (Boston, Mass.)*. 2016;*33*(2):139–150. doi:10.1111/phn.12216.

29. Visser M. Rethinking HIV-prevention for school-going young people based on current behaviour patterns. *Journal of Social Aspects of HIV/AIDS*. 2017;14(1):64-76. doi:10.1080/17290376.2017.1376704.

30. Higgins JP, Thompson SG. Quantifying heterogeneity in a meta- analysis. *Stat Med* 2002;21:1539-58. doi:10.1002/sim.1186.

31. Egger M, Davey Smith G, Schneider M, et al. Bias in meta- analysis detected by a simple, graphical test. *BMJ* 1997;315:629-34. doi:10.1136/bmj.315.7109.629.

For peer review only - http://bmjopen.bmj.com/site/about/guidelines.xhtml

Table 1 Search grid with identified PICO concepts

PICO concepts	
Participants	Youth aged from 15 to 24 years, or the mean age of participants falls in the age range of 15-24 years
Intervention	HIV prevention intervention programme aimed at reducing risky sexual behaviour, including structural, behavioural and combined
Comparators	Control groups who received no intervention or alternative usual interventions, or waitlist controls.
Outcomes	Risky sexual behaviours such as early sexual debut, multiple and/concurrent sexual partnerships, unprotected sex, non-consistent condom use, forced sexual activity, sexual activity under the influence of drugs or alcohol, intergenerational sex, transactional sex etc.

For peer review only

For peer review only - http://bmjopen.bmj.com/site/about/guidelines.xhtml

Table 2 Preliminary search syntax to be used using MEDLINE/PubMed database

Search terms	Query
1	Youth [Text Word] OR Adolescen* [Mesh] OR "Young people" [Text Word] OR Teen* [Text Word] OR "Young adults" [Text Word] OR Students [Text Word]
2	"HIV prevention intervention" [Text Word] OR "HIV prevention strateg*" [Text Word] OR "HIV prevention program*" [Text Word] OR "HIV education prevention program*"[Text Word] OR "Sexual* education program*" [Text Word] OR "Health behav* intervention" [Text Word] OR "Peer* education program*"[Text Word] OR "Peer* HIV prevention intervention" [Text Word] OR "HIV prevention model" [Text Word] OR "HIV/sexually transmitted infection/pregnancy intervention" [Text Word] OR "HIV/AIDS/STI reduction intervention" [Text Word] OR "HIV/STD risk reduction intervention" [Text Word] OR "Behav* intervention" [Text Word] OR "Structural intervention" OR "Behav* intervention" OR "Combin* intervention" OR "Multicomponent intervention" [Text Word] OR "Sexual health intervention" [Text Word] OR "Life skills intervention" [Text Word] OR "HIV/AIDS prevention intervention" [Text Word] OR "HIV/AIDS prevention program*"[Text Word]
3	"Risk* sexual behav*" [Text Word] OR "Unsafe sex" [Text Word] OR "High risk* sex" [Text Word] OR "Unhealthy sexual relations" [Text Word] OR "Sexual health behav*" [Text Word] OR "Sexual risk* behav*" [Text Word] OR "HIV/sexually transmitted infection risk behav*" [Text Word] OR "Sexual risk-taking behav*" [Text Word] OR "Risk* behav* practices" [Text Word] OR "Sexual risk* behav* [Text Word] OR "Risk* behav*" [Text Word] OR "Health risk* behav*" [Text Word] OR "Sexual partners" [Text Word] OR "Sexual behav*" [Text Word]
4	Eff* [Text Word] OR Success [Text Word] OR Outcome [Text Word] OR Impact [Text Word] OR Evaluat* [Text Word] OR "Treatment outcome" [Mesh] OR "Program evaluation" [Mesh]

For peer review only - http://bmjopen.bmj.com/site/about/guidelines.xhtml

5	1 AND 2 AND 3 AND 4 = (Youth [Text Word] OR Adolescen* [Mesh] OR "Young people" [Text Word] OR Teen* [Text Word] OR "Young adults" [Text Word] OR Students [Text Word]) AND ("HIV prevention intervention" [Text Word] OR "HIV prevention strateg*" [Text Word] OR "HIV prevention program*" [Text Word] OR "HIV education prevention program*"[Text Word] OR "Sexual* education program*" [Text Word] OR "Health behav* intervention" [Text Word] OR "Peer* education program*"[Text Word] OR "Peer* HIV prevention intervention" [Text Word] OR "HIV prevention model" [Text Word] OR "HIV/sexually transmitted infection/pregnancy intervention" [Text Word] OR "HIV/AIDS/STI reduction intervention" [Text Word] OR "HIV/STD risk reduction intervention" [Text Word] OR "Behav* intervention" [Text Word] OR "Structural intervention" OR "Behav* intervention" OR "Combin* intervention" OR "Multicomponent intervention" [Text Word] OR "Sexual health intervention" [Text Word] OR "Life skills intervention" [Text Word] OR "HIV/AIDS prevention intervention" [Text Word] OR "HIV/AIDS prevention program*"[Text Word]) AND "Risk* sexual behav*" [Text Word] OR "Unsafe sex" [Text Word] OR "High risk* sex" [Text Word] OR "Unhealthy sexual relations" [Text Word] OR "Sexual health behav*" [Text Word] OR "Sexual risk* behav*" [Text Word] OR "HIV/sexually transmitted infection risk behav*" [Text Word] OR "Sexual risk-taking behav*" [Text Word] OR "Risk* behav* practices" [Text Word] OR "Sexual risk* behav* [Text Word] OR "Risk* behav*" [Text Word] OR "Health risk* behav*" [Text Word] OR "Sexual partners" [Text Word] OR "Sexual behav*" [Text Word]) AND (Eff* [Text Word] OR Success [Text Word] OR Outcome [Text Word] OR Impact [Text Word] OR Evaluat* [Text Word] OR "Treatment outcome" [Mesh] OR "Program evaluation" [Mesh])
	Limits: Publication data from 2011/08/01 to 2021/08/31

For peer review only - http://bmjopen.bmj.com/site/about/guidelines.xhtml

PRISMA-P (Preferred Reporting Items for Systematic review and Meta-Analysis Protocols) 2015 checklist: recommended items to address in a systematic review protocol*

Section and topic	Item No	Checklist item	Page No
**ADMINISTRATIVE INFORMATION**			
Title:			
Identification	1a	Identify the report as a protocol of a systematic review	1
Update	1b	If the protocol is for an update of a previous systematic review, identify as such	
Registration	2	If registered, provide the name of the registry (such as PROSPERO) and registration number	N.A.
Authors:			
Contact	3a	Provide name, institutional affiliation, e-mail address of all protocol authors; provide physical mailing address of corresponding author	1
Contributions	3b	Describe contributions of protocol authors and identify the guarantor of the review	15
Amendments	4	If the protocol represents an amendment of a previously completed or published protocol, identify as such and list changes; otherwise, state plan for documenting important protocol amendments	N.A.
Support:			15
Sources	5a	Indicate sources of financial or other support for the review	
Sponsor	5b	Provide name for the review funder and/or sponsor	
Role of sponsor or funder	5c	Describe roles of funder(s), sponsor(s), and/or institution(s), if any, in developing the protocol	
**INTRODUCTION**			
Rationale	6	Describe the rationale for the review in the context of what is already known	6-7
Objectives	7	Provide an explicit statement of the question(s) the review will address with reference to participants, interventions, comparators, and outcomes (PICO)	7-8
**METHODS**			
Eligibility criteria	8	Specify the study characteristics (such as PICO, study design, setting, time frame) and report characteristics (such as years considered, language, publication status) to be used as criteria for eligibility for the review	8-9, 21
Information sources	9	Describe all intended information sources (such as electronic databases, contact with study authors, trial registers or other grey literature sources) with planned dates of coverage	9
Search strategy	10	Present draft of search strategy to be used for at least one electronic database, including planned limits, such that it could be repeated	10, 22-23
Study records:			11-12

For peer review only

Data management	11a	Describe the mechanism(s) that will be used to manage records and data throughout the review	
Selection process	11b	State the process that will be used for selecting studies (such as two independent reviewers) through each phase of the review (that is, screening, eligibility and inclusion in meta-analysis)	
Data collection process	11c	Describe planned method of extracting data from reports (such as piloting forms, done independently, in duplicate), any processes for obtaining and confirming data from investigators	
Data items	12	List and define all variables for which data will be sought (such as PICO items, funding sources), any pre-planned data assumptions and simplifications	
Outcomes and prioritization	13	List and define all outcomes for which data will be sought, including prioritization of main and additional outcomes, with rationale	12-13
Risk of bias in individual studies	14	Describe anticipated methods for assessing risk of bias of individual studies, including whether this will be done at the outcome or study level, or both; state how this information will be used in data synthesis	11-12,13-14
Data synthesis	15a	Describe criteria under which study data will be quantitatively synthesised	13-14
	15b	If data are appropriate for quantitative synthesis, describe planned summary measures, methods of handling data and methods of combining data from studies, including any planned exploration of consistency (such as I^2, Kendall's τ)	
	15c	Describe any proposed additional analyses (such as sensitivity or subgroup analyses, meta-regression)	
	15d	If quantitative synthesis is not appropriate, describe the type of summary planned	13-14
Meta-bias(es)	16	Specify any planned assessment of meta-bias(es) (such as publication bias across studies, selective reporting within studies)	
Confidence in cumulative evidence	17	Describe how the strength of the body of evidence will be assessed (such as GRADE)	11-12

*** It is strongly recommended that this checklist be read in conjunction with the PRISMA-P Explanation and Elaboration (cite when available) for important clarification on the items. Amendments to a review protocol should be tracked and dated. The copyright for PRISMA-P (including checklist) is held by the PRISMA-P Group and is distributed under a Creative Commons Attribution Licence 4.0.**

From: Shamseer L, Moher D, Clarke M, Ghersi D, Liberati A, Petticrew M, Shekelle P, Stewart L, PRISMA-P Group. Preferred reporting items for systematic review and meta-analysis protocols (PRISMA-P) 2015: elaboration and explanation. BMJ. 2015 Jan 2;349(jan02 1):g7647.

For peer review only

BMJ Open

A systematic review protocol of the effectiveness of HIV prevention interventions for reducing risky sexual behaviour among youth globally

Journal:	*BMJ Open*
Manuscript ID	bmjopen-2021-056929.R1
Article Type:	Protocol
Date Submitted by the Author:	15-Mar-2022
Complete List of Authors:	Mbengo, Fungai; Edith Cowan University, School of Nursing and Midwifery<br>Zgambo, Maggie; Edith Cowan University, School of Nursing and Midwifery<br>Afrifa-Yamoah, Ebenezer; Edith Cowan University, School of Science<br>Kalembo, Fatch; Curtin University, School of Nursing, Midwifery and Paramedicine<br>Honda, Takanori ; Kyushu University, Department of Epidemiology and Public Health, Graduate School of Medical Sciences<br>Shinpuku, Yoko ; Hiroshima University, Department of Global Health Nursing, Division of Nursing Sciences, Graduate School of Biomedical and Health Sciences<br>Chen, Sanmei; Hiroshima University, Department of Global Health Nursing, Division of Nursing Sciences, Graduate School of Biomedical and Health Sciences
**Primary Subject Heading**:	Infectious diseases
Secondary Subject Heading:	HIV/AIDS, Infectious diseases, Public health, Global health
Keywords:	HIV & AIDS < INFECTIOUS DISEASES, Public health < INFECTIOUS DISEASES, Infection control < INFECTIOUS DISEASES, PREVENTIVE MEDICINE

SCHOLARONE™
Manuscripts

A systematic review protocol of the effectiveness of HIV prevention interventions for reducing risky sexual behaviour among youth globally

Fungai Mbengo[1], Maggie Zgambo[1], Ebenezer Afrifa-Yamoah[2], Fatch Welcome Kalembo[3], Takanori Honda[4], Yoko Shimpuku[5], Sanmei Chen[†5]

Affiliations:

[1] School of Nursing and Midwifery, Edith Cowan University, Western Australia, Australia

[2] School of Science, Edith Cowan University, Western Australia, Australia

[3] School of Nursing, Midwifery and Paramedicine, Curtin University, Western Australia, Australia

[4] Department of Epidemiology and Public Health, Graduate School of Medical Sciences, Kyushu University, Fukuoka, Japan

[5] Department of Global Health Nursing, Division of Nursing Sciences, Graduate School of Biomedical and Health Sciences, Hiroshima University, Hiroshima Japan

[†]Corresponding author:

Dr. Sanmei Chen

Department of Global Health Nursing, Division of Nursing Sciences,

Graduate School of Biomedical and Health Sciences, Hiroshima University

734-8553, 1-2-3 Kasumi, Minami Ward, Hiroshima City, Japan

For peer review only

For peer review only - http://bmjopen.bmj.com/site/about/guidelines.xhtml

TEL: +8182-257-5377 Email: chens@hiroshima-u.ac.jp

ORCID: 0000-0003-0811-1701

Authors' email address:

Fungai Mbengo: fmbengo@our.ecu.edu.au

Maggie Zgambo: m.zgambo@ecu.edu.au

Ebenezer Afrifa-Yamoah: e.afrifayamoah@ecu.edu.au

Fatch Welcome Kalembo: fatchwelcom.kalembo@curtin.edu.au

Takanori Honda: honda.takanori.597@m.kyushu-u.ac.jp

Yoko Shimpuku: yokoshim@hiroshima-u.ac.jp

Sanmei Chen: chens@hiroshima-u.ac.jp

For peer review only

For peer review only - http://bmjopen.bmj.com/site/about/guidelines.xhtml

ABSTRACT

Introduction HIV prevention interventions focused at reducing risky sexual behaviours are an important strategy for preventing Human Immunodeficiency Virus (HIV) infection among youth (15-24 years) who continue to be vulnerable to the disease. This systematic review aims to synthesise current global evidence on the effectiveness of HIV prevention interventions for reducing risky sexual behaviour among youth in the last decade.

Methods and analysis MEDLINE/PubMed, EMBASE, PsychINFO, ProQuest Central, CINAHL and Web of Science databases, ClinicalTrials.gov and the World Health Organization International Clinical Trials Registry Platform, and reference lists of included studies and systematic reviews on effectiveness of HIV prevention interventions for reducing risky sexual behaviour among youth will be searched for articles published from August 2011 to August 2021. Eligible studies will be longitudinal studies including randomised controlled trials and quasi-experimental studies that examined the effectiveness of HIV prevention interventions among youth populations (15-24 years) with risky sexual behaviour as a primary or secondary outcome. Study selection and quality assessment will be undertaken independently by three reviewers, and disagreements will be resolved through consensus. Data analysis will be undertaken using RevMan software version 5.3.3. A random effects meta-analysis will be conducted to report heterogeneous data where statistical pooling is achievable. We will use I^2 statistics to test for heterogeneity. Where appropriate, a funnel plot will be generated to assess publication bias. Where statistical pooling is unachievable, the

For peer review only - http://bmjopen.bmj.com/site/about/guidelines.xhtml

findings will be reported in a narrative form, together with tables and figures to assist in data presentation if required. Reporting of the systematic review will be informed by Preferred Reporting Items for Systematic Reviews and Meta-Analyses (PRISMA) guidelines.

Ethics and dissemination Ethical approval is not required. Findings of the systematic review will be published in a peer-reviewed journal. The findings will be of interest to researchers, healthcare practitioners and policy makers.

PROSPERO registration number CRD42021271774

For peer review only

For peer review only - http://bmjopen.bmj.com/site/about/guidelines.xhtml

Strengths and limitations of this study

- This systematic review protocol follows the Preferred Reporting Items for Systematic Review and Meta-analysis Protocols guidelines with transparency to the methods and processes that will be used.
- This systematic review will address a gap in the current global evidence by comprehensively including research findings worldwide on the effectiveness of HIV prevention interventions for reducing risky sexual behaviour among youth in the last decade.
- This systematic review will be one of the few to focus on behavioural-level, structural-level and combined interventions for HIV prevention targeting youth.
- This systematic review will consider only publications written in English. This limitation will result in language bias.
- The measures of risky sexual behaviour may vary between studies. The variations may create bias and render pooled of estimates meaningless.

For peer review only

For peer review only - http://bmjopen.bmj.com/site/about/guidelines.xhtml

INTRODUCTION

According to the Joint United Nations Programme on HIV/AIDS (UNAIDS), 38 million people were living with Human Immunodeficiency Virus (HIV) in 2019 and about 35 million had died since HIV was first recognized globally.[1] Over the last decade, there has been a remarkable reduction in HIV infections, HIV related mortality and morbidity in the general population globally following the introduction of the universal antiretroviral therapy.[2] However, recent reports indicate that HIV remains a leading cause of death among the youth population across Sub-Saharan Africa, where 80% of young people living with HIV reside.[1 3] Furthermore, in 2019, one third of the global HIV infections occurred among youth aged between 15 and 24 years and the infection rate in this population is anticipated to increase yearly by 13% leading to an estimation of 3.5 million new infections by 2030.[4 5]

Youth are vulnerable to HIV because of a myriad of factors that influence risky sexual behaviours including low self-esteem, poverty, peer pressure[6], alcohol or drug abuse[7], cultural practices, gender-disparities[8 9], limited HIV-related knowledge and gender-based violence.[10] According to the socio-ecological model, these factors affect youth at individual, interpersonal, community, institutional and structural levels.[11 12] This model has been used to guide the development of HIV prevention interventions aimed at reducing risky sexual behaviours among youth.[12-15] In this review, risky sexual behaviours are activities engaged by an individuals that result in negative outcome and these activities are engaging in transactional sex, unprotected sex, multiple sex

For peer review only - http://bmjopen.bmj.com/site/about/guidelines.xhtml

partners, intergenerational sex, having partners who are at risk of HIV infection such as using injectable drugs, early sex debut and early marriages.[16,17,18]

HIV prevention interventions for reducing risky sexual behaviours can be classified as either structural, behavioural or combined.[13-15] Structural interventions aim at reducing risky sexual behaviour by addressing structural-level factors such as poverty, unemployment, limited access to education and social norms.[13-15] The behavioural interventions facilitate risky sexual behaviour reduction by targeting individual-level factors such as knowledge, attitudes, self-esteem and self-efficacy.[13-15] Lastly, the combined interventions reduce risky sexual behaviour by addressing one or more individual-level factors, as well as one or more structural-level factors.[13-15]

In the last decade, researchers have conducted systematic reviews on structural, behavioural or combined HIV prevention interventions targeting youth.[13 19-24] However, these reviews have synthesised research findings from studies that were conducted in a specific region, for instance, Sub-Saharan Africa[13 19 20], developing countries[21 22], middle-income countries[23], or a specific country.[24] One review has particularly focused on the female gender.[13] Most of these reviews have focused on behavioural interventions only.[19 21-23] Synthesising global evidence on the subject has potential to identify more effective interventions for reducing risky sexual behaviours among youth.

Following this background, we propose to undertake a systematic review and meta-analysis to synthesise current global evidence on the effectiveness of HIV prevention interventions for reducing risky sexual behaviours among youth in the last

For peer review only

For peer review only - http://bmjopen.bmj.com/site/about/guidelines.xhtml

decade. Specifically, this systematic review and meta-analysis seeks to answer the following questions:1) What HIV prevention interventions are effective for reducing risky sexual behaviour among youth? 2) What is the level of effectiveness of interventions designed to reduce risky sexual behaviour among youth? 3) What factors affect the effectiveness of the identified interventions? To prevent duplication of reviews, a preliminary search of similar protocols or reviews was conducted in July 2021 in CINAHL, Cochrane Library, MEDLINE, Google Scholar, The International Prospective Register of Systematic Reviews (PROSPERO) and Joanna Briggs Institute (JBI) databases. No review protocol or systematic review on this topic published in the last decade was identified.

METHODS AND ANALYSIS

This will be a systematic review and meta-analysis designed to analyse recent global evidence on the effectiveness of HIV prevention interventions for reducing risky sexual behaviour among youth. This review will commence in April 2022. This protocol has been developed in accordance with the Preferred Reporting Items for Systematic Review and Meta-analysis Protocols (PRISMA-P) guidelines.[25] Reporting of the synthesised findings will be informed by PRISMA guidelines.[26] This protocol has been registered in the PROSPERO. Important amendments to this protocol will be published along with the results of the systematic review.

Eligibility criteria

For peer review only - http://bmjopen.bmj.com/site/about/guidelines.xhtml

To be eligible for this review, studies will need to report on youth aged from 15 to 24 years and be conducted in either clinical or non-clinical setting. Inclusion of studies with participants aged within and outside the identified age bracket will depend on the mean age that falls within the age range of 15-24 years. For recency, all studies conducted from August 2011 to August 2021 will be considered. The review will include studies conducted in any geographical location. This review will include the following 3 types of intervention: 1) behavioural interventions that facilitate reduction of risky sexual behaviour by addressing individual-level factors; 2) structural interventions that facilitate reduction of risky sexual behaviour by targeting structural-level factors; 3) combined interventions that facilitate reduction of risky sexual behaviour by targeting at least one individual-level factor and at least one structural-level factor. Studies that do not measure risky sexual behaviour as a primary or secondary outcome will be excluded. In the source trials, the comparators could be control groups who received no intervention or alternative usual interventions, or waitlist controls. The review will consider only longitudinal studies such as quasi-experimental studies, randomized controlled trials and cluster-randomized trials and non-randomized controlled trials. Only publications written in English will be considered.

Information sources

We will electronically search the following databases to retrieve relevant articles: MEDLINE/PubMed, EMBASE, PsychINFO, ProQuest Central, CINAHL and Web of Science databases. We will also search ClinicalTrials.gov and the World Health Organization International Clinical Trials Registry Platform to identify ongoing or

For peer review only - http://bmjopen.bmj.com/site/about/guidelines.xhtml

unpublished eligible trials. To maximise the search for relevant articles, we will review reference lists of included studies and systematic reviews on effectiveness of HIV prevention interventions for reducing risky sexual behaviour among youth.

Search strategy

The Population, Intervention, Comparator and Outcome (PICO) Model as recommended by JBI will be used to develop a search grid for this review. In this review, the participants are youth aged from 15 to 24 years of age; interventions are HIV prevention interventions; comparators are control groups, and outcomes are risky sexual behaviours. The search grid with identified PICO concepts is presented in online supplementary file Table 1.

Using the identified PICO concepts, a three-step search strategy will be utilised to identify relevant studies. First, keywords for PICO concepts will be brainstormed by reviewers before undertaking an initial limited search of PICO concepts in the MEDLINE/PubMed database to identify controlled vocabulary Medical Subject Headings (MeSH). A second search of EMBASE, MEDLINE/PubMed, PsychINFO, ProQuest Central, CINAHL and Web of Science databases, ClinicalTrials.gov and the World Health Organization International Clinical Trials Registry Platform will be conducted using the identified keywords and MeSH terms. Third, we will use these information sources to find other relevant keywords and terms. Boolean operators such as ‘OR’ and ‘AND’ will be applied when combining similar search terms and different search terms, respectively. For example, (youth OR young people OR teen OR young adults OR adolescents) AND (HIV prevention intervention OR HIV prevention strateg* OR HIV

For peer review only - http://bmjopen.bmj.com/site/about/guidelines.xhtml

prevention program* OR HIV education prevention program* OR Sexual* education program*). A detailed search strategy is presented in online supplementary file Table S1, using MEDLINE/PubMed as an example. The search strategy will be adapted to other information sources.

Study selection

Following the search, all identified citations will be collated and uploaded into EndNote X9 Reference Management System. Duplicates will be removed before importing the references into the Covidence online systematic review tool as recommended by the Cochrane Collaboration.[27] This tool is designed to assist reviewers to screen abstracts and full texts of identified articles, risk of bias assessment and data extraction.[27] In this review, however, this tool will only be used to select eligible studies. Abstracts of the relevant full texts will be assessed for eligibility by FM, MZ and SC, independently. Full-text articles for the selected titles will be further reviewed independently by these reviewers. Following this selection, methodological quality of each included study will be independently assessed by three reviewers using the JBI Critical Appraisal Checklist for Randomized Controlled Trials and JBI Critical Appraisal Checklist for Quasi-Experimental Studies.[28] These two appraisal tools include 12 and 9 criteria (rating: yes, no, unclear), respectively, with a narrative form for decision making. Two reviewers will rate each study as a JBI score ranging from 1 to the total score (12 or 9), with higher score indicating higher quality. Studies that meet less than 50% of all criteria will be excluded. Each reviewer will leave an audit trail with reasons for each decision undertaken, which will later be compared across all the reviewers. Cohen's kappa

For peer review only - http://bmjopen.bmj.com/site/about/guidelines.xhtml

coefficient will be used to measure agreement among the reviewers. Any disagreements that arise among the reviewers at each stage of the study selection process will be resolved through discussions to reach a consensus. The results of the search will be illustrated following the PRISMA flow diagram.[26]

Assessment of risk of bias and quality of evidence

As mentioned above, three reviewers will independently rate the quality of each included study using the JBI Critical Appraisal Checklist for Randomized Controlled Trials and JBI Critical Appraisal Checklist for Quasi-Experimental Studies.[28] In addition, we will examine the quality of evidence for each outcome by using the GRADE approach,[29] because the quality of evidence often varies between outcomes.[30] We will not exclude any study on the basis of the GRADE score. Authors of papers will be contacted to request missing information that is necessary for appraisal.

Data extraction

Data will be extracted by FM using the standardised JBI data extraction tool.[28] MZ and SC will verify the extracted data. Details extracted will include: 1) characteristics of the study: author, year of publication, the title of the study, study objective, study location, setting, 2) methodological characteristics: study design, research questions and/or hypotheses, study population, sample characteristics, groups and controls, type of intervention, length of intervention, delivery mode, theoretical framework, and length of follow-up, measurements, data analyses; 3) main findings and conclusions. Authors will

For peer review only - http://bmjopen.bmj.com/site/about/guidelines.xhtml

be contacted to request for full articles if only abstracts are accessible and for information if the main outcome data and methods are missing or unclear.

Outcomes and prioritisation

The primary outcome of this review is risky sexual behaviour, which will be defined as engaging in one or more of the following sexual behaviours as derived from literature: 1) having first sexual activity at or before the age of 15 (early sexual debut), 2) engaging in sexual activity without a condom, 3) inconsistent condom use, 4) having multiple sex partners, 5) intergenerational sex, 6) transactional sex 7) forced sex, 8) early marriages, 9) having partners who are at risk of HIV infection such as using injectable drugs and 10) having been pregnant or fathered a child at a younger age (18 years or younger). [17 31 32] The secondary outcome measures will be sexually transmitted diseases (STDs) rates, pregnancy rates, birth rates and changes in mediating factors that affect risky sexual behaviours such as knowledge, attitudes and beliefs. Interventions will be deemed effective if the frequency of behaviour is reduced or stopped and if there is a positive change in the prevalence of the secondary outcomes and mediating factors. Factors that affect the effectiveness of the intervention such as study design, sample characteristics, intervention content, duration of intervention, the dosage of intervention, length of follow-up and theoretical framework will also be examined.

Data synthesis

Statistical analyses will be conducted using RevMan software (version 5.3.3; The Cochrane Collaboration). We will perform analyses for all outcomes on an intention to

For peer review only - http://bmjopen.bmj.com/site/about/guidelines.xhtml

treat basis. Effect sizes expressed as odds ratio (for dichotomous data) and weighted mean differences or standardised mean differences (for continuous data) and their 95% confidence intervals will be calculated for analysis. A *p*-value of less than 0.05 will be considered as statistically significant. Heterogeneity will be assessed statistically using the Chi-squared test and the I-squared index.[33] We will use fixed effects models to pool outcomes if significant heterogeneity is not present (I^2 <50%). We will use random effects models when significant heterogeneity is present (I^2 ≥50%). If sufficient data is available, subgroup analyses will be conducted to explore the heterogeneity between the studies. A funnel plot will be generated to assess publication bias if there are 10 or more studies included in a meta-analysis. Statistical tests for funnel plot asymmetry (Egger test, Begg test, Harbord test) will be performed where appropriate. We will analyse the data separately for each of the 3 types of interventions (i.e., structural, behavioural, or combined interventions). Several subgroup analyses will be conducted based on study setting, year of publication, length of follow-up and so on. We will also conduct sensitivity analyses to test the robustness of our findings, such as by excluding quasi-randomised trials. Where statistical pooling is unachievable[34], the findings will be reported in a narrative form, together with tables and figures to assist in data presentation if required.

For peer review only - http://bmjopen.bmj.com/site/about/guidelines.xhtml

Table 1: Search grid with identified PICO concepts

PICO concepts	
Participants	Youth aged from 15 to 24 years, or the mean age of participants falls in the age range of 15-24 years
Intervention	HIV prevention intervention programme aimed at reducing risky sexual behaviour, including structural, behavioural and combined
Comparators	Control groups who received no intervention or alternative usual interventions, or waitlist controls.
Outcomes	Risky sexual behaviours: 1) having first sexual activity at or before the age of 15 (early sexual debut), 2) engaging in sexual activity without a condom, 3) inconsistent condom use, 4) having multiple sex partners, 5) intergenerational sex, 6) transactional sex 7) forced sex, 8) early marriages, 9) having partners who are at risk of HIV infection sich as using injectable drugs and 10) having been pregnant or fathered a child at a younger age (18 years or younger.

For peer review only - http://bmjopen.bmj.com/site/about/guidelines.xhtml

Patient and public involvement

No patient involved.

Ethics and dissemination

Ethical approval is not required for a systematic review. Findings of the systematic review will be disseminated through publication in a peer reviewed journal. The findings will be of interest to researchers, healthcare practitioners and policy makers.

Author contributions FM, MA, and SC conceptualized and designed the protocol, and drafted the manuscript. EAY, FWK, TH and YS contributed to the plan of search strategy, data extraction, and data analysis. All authors contributed to the manuscript revision.

Funding SM was supported by Grants-in-Aid for Early-Career Scientists (JP19K19474) from the Ministry of Education, Culture, Sports, Science and Technology of Japan. The funding source had no role in the study conception, the preparation of the manuscript, or the decision to submit the manuscript for publication.

Competing interests We have no competing interests to declare.

For peer review only

For peer review only - http://bmjopen.bmj.com/site/about/guidelines.xhtml

References

1. UNAIDS AIDSinfo: people living with HIV receiving ART. https://aidsinfo.unaids.org/ (accessed 6 Jun 2021).
2. Teeraananchai S, Kerr S, Amin J, et al. Life expectancy of HIV-positive peopleafter starting combination antiretroviral therapy: a meta-analysis. *HIV medicine* 2017;18(4):256-66.
3. Kharsany ABM, Karim QA. HIV Infection and AIDS in Sub-Saharan Africa: Current Status, Challenges and Opportunities. *The open AIDS journal* 2016;10:34-48.
4. United Nations Children's Fund. Children and AIDS. Statistical Update. (2017).
5. UNAIDS. AIDSinfo: Global data on HIV epidemiology and response http://aidsinfo.unaids.org/ (accessed 29 Aug 2021).
6. DiClemente RJ, Crittenden CP, Rose E, et al. Psychosocial predictors of HIV-associated sexual behaviors and the efficacy of prevention interventions in adolescents at-risk for HIV infection: what works and what doesn't work? *Psychosomatic Medicine* 2008;70(5):598-605.
7. Govender K, Cowden RG, Asante KO, et al. Sexual risk behavior: A multi-system model of risk and protective factors in South African adolescents. *Prevention Science* 2019;20(7):1054-65.
8. Dancy BL, Jere DL, Kachingwe SI, et al. HIV risk reduction intervention for rural adolescents in Malawi. *Journal of HIV/AIDS & social services* 2014;13(3):271-91.

For peer review only - http://bmjopen.bmj.com/site/about/guidelines.xhtml

9. Van Staden A, Badenhorst G. Reviewing gender and cultural factors associated with HIV/AIDS among university students in the South African context. *Curationis* 2009;*32*(4):19-28.

10. Harper GW, Riplinger AJ, Neubauer LC, et al. Ecological factors influencing HIV sexual risk and resilience among young people in rural Kenya: Implications for prevention. *Health education research* 2014;29(1):131-46.

11. Khuzwayo N,Taylor M. Exploring the socio-ecological levels for prevention of sexual risk behaviours of the youth in uMgungundlovu District Municipality, KwaZulu-Natal. *African journal of primary health care & family medicine* 2018;10(1):e1-e8.

12. Lee BC, Bendixsen C, Liebman AK, et al. Using the Socio-Ecological Model to Frame Agricultural Safety and Health Interventions. *Journal of Agromedicine* 2017;22(4):298-303.

13. Muthoni CN, Kneipp SM, Gichane MW, et al. A systematic review of HIV interventions for young women in sub-Saharan Africa. *AIDS and Behavior* 2020;24:3395-413.

14. Pettifor A, Bekker LG, Hosek S, et al. Preventing HIV among young people: research priorities for the future. *Journal of acquired immune deficiency syndromes (1999)* 2013;63(0 2):S155.

15. Hosek S, Pettifor A. HIV Prevention Interventions for Adolescents. *Current HIV/AIDS reports* 2019;16(1):120-8.

For peer review only - http://bmjopen.bmj.com/site/about/guidelines.xhtml

16. Zgambo, M., Kalembo, F. W., & Mbakaya, B. C. Risky behaviours and their correlates among adolescents living with HIV in sub-Saharan Africa: A systematic review. *Reproductive Health* 2018;15(1):180.

17. Zgambo, M., Arabiat, D., & Ireson, D. “We just do it...we are dead already”: Exploring the sexual behaviors of youth living with HIV. *Journal of Adolescence* 2022:1–11.

18. Pringle, J., Mills, K. L., McAteer, J., Jepson, R., Hogg, E., Anand, N., & Blakemore, S. J. The physiology of adolescent sexual behaviour: A systematicreview. *Cogent Social Sciences* 2017;3(1):1368858.

19. Mwale M, Muula AS. Systematic review: a review of adolescent behavior change interventions [BCI] and their effectiveness in HIV and AIDS prevention in sub-Saharan Africa. *BMC public health* 2017;17(1):1-9.

20. Sani AS, Abraham C, Denford S, et al. School-based sexual health education interventions to prevent STI/HIV in sub-Saharan Africa: a systematic review and meta-analysis. *BMC public health* 2016;16(1):1-26.

21. Dei Jnr LA. The efficacy of HIV and sex education interventions among youths in developing countries: A review. *Scientific & Academic Publishing* 2016:6(1):1-17.

22. Ibrahim S, Sidani S. Community based HIV prevention intervention in developing countries: a systematic review. *Advances in Nursing* 2014:174960.

23. Fonner VA, Armstrong KS, Kennedy CE, et al. School Based Sex Education and HIV Prevention in Low and Middle-Income Countries: A Systematic Review and Meta-Analysis. *Plos one* 2014;9(3):e89692.

For peer review only - http://bmjopen.bmj.com/site/about/guidelines.xhtml

24. Scott-Sheldon AJL, Walstrom P, Harrison AC, et al. Sexual risk reduction interventions for HIV prevention among South African Youth: a meta-analytic review. *Current HIV research* 2013;11(7):549-58.
25. Shamseer L, Moher D, Clarke M, et al. Preferred reporting items for systematic review and meta-analysis protocols (PRISMA-P) 2015: elaboration and explanation. *BMJ* 2015;349:g7647.
26. Moher D, Liberati A, Tetzlaff J, et al. Preferred reporting items for systematic reviews and meta-analyses: the PRISMA statement. *PLoS medicine* 2009;6(7):e1000097.
27. Coevidence. How to conduct a systematic review from beginning to end. https://www.covidence.org/blog/how-to-conduct-a-systematic-review-from beginning-to end/?campaignid=13271466385&adgroupid=123024099579&adid=524233276792&gclid=Cj0KCQjw0KHBhDDARIsAFJ6UGg4C_yy8JcRM0TnV0yUnCjPInKnkngt39SX10gyJ_Xyszbo9SWyfQYaAmTWEALw_wcB (accessed 12 Jul 2021).
28. Aromataris E, Munn Z, eds. Joanna Briggs Institute Reviewer's Manual [Internet]. Adelaide: Joanna Briggs Institute. https://reviewersmanual.joannabriggs.org/ (accessed 26 April 2020).
29. Guyatt GH, Oxman AD, Vist GE, et al. GRADE: an emerging consensus on rating quality of evidence and strength of recommendations. *BMJ* 2008;336:924-6.
30. Balshem H, Helfand M, Schunemann HJ, et al. GRADE guidelines: 3. Rating the quality of evidence. *Journal of clinical epidemiology*. 2011;64(4):401-6.

For peer review only - http://bmjopen.bmj.com/site/about/guidelines.xhtml

31. Brawner BM, Alexander KA, Fannin EF, et al. The role of sexual health professionals in developing a shared concept of risky sexual behavior as it relates to HIV transmission. *Public health nursing (Boston, Mass.)*. 2016;*33*(2):139–150.

32. Visser M. Rethinking HIV-prevention for school-going young people based on current behaviour patterns. *Journal of Social Aspects of HIV/AIDS*. 2017;14(1):64-76.

33. Higgins JP, Thompson SG. Quantifying heterogeneity in a meta- analysis. *Stat Med* 2002;21:1539-58.

34. Egger M, Davey Smith G, Schneider M, et al. Bias in meta- analysis detected by a simple, graphical test. *BMJ* 1997;315:629-34.

For peer review only

For peer review only - http://bmjopen.bmj.com/site/about/guidelines.xhtml

Supplementary information

Table S1: Search strategy

Theme	Number	Search terms
**participants**	#1	Youth [Text Word] OR Adolescen* [Mesh Term] OR "Young people" [Text Word] OR Teen* [Text Word] OR "Young adults" [Text Word] OR Students [Text Word]
**intervention**	#2	"HIV prevention intervention" [Text Word] OR "HIV prevention strateg*" [Text Word] OR "HIV prevention program*" [Text Word] OR "HIV education prevention program*"[Text Word] OR "Sexual* education program*" [Text Word] OR "Health behav* intervention" [Text Word] OR "Peer* education program*"[Text Word] OR "Peer* HIV prevention intervention" [Text Word] OR "HIV prevention model" [Text Word] OR "HIV/sexually transmitted infection/pregnancy intervention" [Text Word] OR "HIV/AIDS/STI reduction intervention" [Text Word] OR "HIV/STD risk reduction intervention" [Text Word] OR "Behav* intervention" [Text Word] OR "Structural intervention" OR "Behav* intervention" OR "Combin* intervention" OR "Multicomponent intervention" [Text Word] OR "Sexual health intervention" [Text Word] OR "Life skills intervention" [Text Word] OR "HIV/AIDS prevention intervention" [Text Word] OR "HIV/AIDS prevention program*"[Text Word]
**outcomes**	#3	"Risk* sexual behav*" [Text Word] OR "Unsafe sex" [Text Word] OR "High risk* sex" [Text Word] OR "Unhealthy sexual relations" [Text Word] OR "Sexual health behav*" [Text Word] OR "Sexual risk* behav*" [Text Word] OR "HIV/sexually transmitted infection risk behav*" [Text Word] OR "Sexual risk-taking behav*" [Text Word] OR "Risk* behav* practices" [Text Word] OR "Risk* behav*"

For peer review only

		[Text Word] OR "Health risk* behav*" [Text Word] OR "Sexual partners" [Text Word] OR "Sexual behav*" [Text Word] OR "Forced sex" [Text Word] OR "Coerced sex" [Text Word] OR "Early sexual debut" [Text Word] OR "Early marriage" [Text Word] OR "Transactional sex" [Text Word] OR "Intergenerational sex" [Text Word] OR Multiple sexual partners [MeSH term] OR "Sex partners" [Text Word] OR "Multiple sex partners" OR "Concurrent sexual partners" [Text Word] OR "Condomless sex" [Text Word] OR "Unprotected sex" [Text Word] OR "Inconsistent condom use" [Text Word] OR "Pregnancy incidence" [Text Word]
**Search**	#4	1 AND 2 AND 3 = (Youth [Text Word] OR Adolescen* [Mesh Term] OR "Young people" [Text Word] OR Teen* [Text Word] OR "Young adults" [Text Word] OR Students [Text Word]) AND ("HIV prevention intervention" [Text Word] OR "HIV prevention strateg*" [Text Word] OR "HIV prevention program*" [Text Word] OR "HIV education prevention program*"[Text Word] OR "Sexual* education program*" [Text Word] OR "Health behav* intervention" [Text Word] OR "Peer* education program*"[Text Word] OR "Peer* HIV prevention intervention" [Text Word] OR "HIV prevention model" [Text Word] OR "HIV/sexually transmitted infection/pregnancy intervention" [Text Word] OR "HIV/AIDS/STI reduction intervention" [Text Word] OR "HIV/STD risk reduction intervention" [Text Word] OR "Behav* intervention" [Text Word] OR "Structural intervention" OR "Behav* intervention" OR "Combin* intervention" OR "Multicomponent intervention" [Text Word] OR "Sexual health intervention" [Text Word] OR "Life skills intervention" [Text Word] OR "HIV/AIDS

For peer review only - http://bmjopen.bmj.com/site/about/guidelines.xhtml

		prevention intervention" [Text Word] OR "HIV/AIDS prevention program*"[Text Word]) AND "Risk* sexual behav*" [Text Word] OR "Unsafe sex" [Text Word] OR "High risk* sex" [Text Word] OR "Unhealthy sexual relations" [Text Word] OR "Sexual health behav*" [Text Word] OR "Sexual risk* behav*" [Text Word] OR "HIV/sexually transmitted infection risk behav*" [Text Word] OR "Sexual risk-taking behav*" [Text Word] OR "Risk* behav* practices" [Text Word] OR "Risk* behav*" [Text Word] OR "Health risk* behav*" [Text Word] OR "Sexual partners" [Text Word] OR "Sexual behav*" [Text Word] OR "Forced sex" [Text Word] OR "Coerced sex" [Text Word] OR "Early sexual debut" [Text Word] OR "Early marriage" [Text Word] OR "Transactional sex" [Text Word] OR "Intergenerational sex" [Text Word] OR Multiple sexual partners [MeSH term] OR "Sex partners" [Text Word] OR "Multiple sex partners" OR "Concurrent sexual partners" [Text Word] OR "Condomless sex" [Text Word] OR "Unprotected sex" [Text Word] OR "Inconsistent condom use" [Text Word] OR "Pregnancy incidence" [Text Word]
**Limits**	#5	Publications from 2011/08/01 to 2021/08/31<br>Age outside 15 to 24 years<br>Non-English publications

For peer review only

PRISMA-P (Preferred Reporting Items for Systematic review and Meta-Analysis Protocols) 2015 checklist: recommended items to address in a systematic review protocol*

Section and topic	Item No	Checklist item	Page No
**ADMINISTRATIVE INFORMATION**			
Title:			
Identification	1a	Identify the report as a protocol of a systematic review	1
Update	1b	If the protocol is for an update of a previous systematic review, identify as such	
Registration	2	If registered, provide the name of the registry (such as PROSPERO) and registration number	N.A.
Authors:			
Contact	3a	Provide name, institutional affiliation, e-mail address of all protocol authors; provide physical mailing address of corresponding author	1
Contributions	3b	Describe contributions of protocol authors and identify the guarantor of the review	16
Amendments	4	If the protocol represents an amendment of a previously completed or published protocol, identify as such and list changes; otherwise, state plan for documenting important protocol amendments	N.A.
Support:			16
Sources	5a	Indicate sources of financial or other support for the review	
Sponsor	5b	Provide name for the review funder and/or sponsor	
Role of sponsor or funder	5c	Describe roles of funder(s), sponsor(s), and/or institution(s), if any, in developing the protocol	
**INTRODUCTION**			
Rationale	6	Describe the rationale for the review in the context of what is already known	6-7
Objectives	7	Provide an explicit statement of the question(s) the review will address with reference to participants, interventions, comparators, and outcomes (PICO)	7-8, Table 1
**METHODS**			
Eligibility criteria	8	Specify the study characteristics (such as PICO, study design, setting, time frame) and report characteristics (such as years considered, language, publication status) to be used as criteria for eligibility for the review	8-9, Table 1
Information sources	9	Describe all intended information sources (such as electronic databases, contact with study authors, trial registers or other grey literature sources) with planned dates of coverage	9, Table S1
Search strategy	10	Present draft of search strategy to be used for at least one electronic database, including planned limits, such that it could be repeated	10, Table S1

For peer review only

Study records:			11-12
Data management	11a	Describe the mechanism(s) that will be used to manage records and data throughout the review	
Selection process	11b	State the process that will be used for selecting studies (such as two independent reviewers) through each phase of the review (that is, screening, eligibility and inclusion in meta-analysis)	
Data collection process	11c	Describe planned method of extracting data from reports (such as piloting forms, done independently, in duplicate), any processes for obtaining and confirming data from investigators	
Data items	12	List and define all variables for which data will be sought (such as PICO items, funding sources), any pre-planned data assumptions and simplifications	
Outcomes and prioritization	13	List and define all outcomes for which data will be sought, including prioritization of main and additional outcomes, with rationale	12-13
Risk of bias in individual studies	14	Describe anticipated methods for assessing risk of bias of individual studies, including whether this will be done at the outcome or study level, or both; state how this information will be used in data synthesis	11-12,13-14
Data synthesis	15a	Describe criteria under which study data will be quantitatively synthesised	13-14
	15b	If data are appropriate for quantitative synthesis, describe planned summary measures, methods of handling data and methods of combining data from studies, including any planned exploration of consistency (such as I^2, Kendall's τ)	
	15c	Describe any proposed additional analyses (such as sensitivity or subgroup analyses, meta-regression)	
	15d	If quantitative synthesis is not appropriate, describe the type of summary planned	13-14
Meta-bias(es)	16	Specify any planned assessment of meta-bias(es) (such as publication bias across studies, selective reporting within studies)	14
Confidence in cumulative evidence	17	Describe how the strength of the body of evidence will be assessed (such as GRADE)	11-12

*** It is strongly recommended that this checklist be read in conjunction with the PRISMA-P Explanation and Elaboration (cite when available) for important clarification on the items. Amendments to a review protocol should be tracked and dated. The copyright for PRISMA-P (including checklist) is held by the PRISMA-P Group and is distributed under a Creative Commons Attribution Licence 4.0.**

From: Shamseer L, Moher D, Clarke M, Ghersi D, Liberati A, Petticrew M, Shekelle P, Stewart L, PRISMA-P Group. Preferred reporting items for systematic review and meta-analysis protocols (PRISMA-P) 2015: elaboration and explanation. BMJ. 2015 Jan 2;349(jan02 1):g7647.

For peer review only
